# Drivers of district-level differences in outpatient antibiotic prescribing in Germany: a qualitative study with prescribers

**DOI:** 10.1186/s12913-024-11059-z

**Published:** 2024-05-06

**Authors:** Benjamin Schüz, Oliver Scholle, Ulrike Haug, Roland Tillmann, Christopher Jones

**Affiliations:** 1 Institute of Public Health and Nursing Research, University of Bremen, Bremen, Germany; 2 Department of Clinical Epidemiology, Leibniz Institute of Prevention Research and Epidemiology – BIPS, Bremen, Germany; 3 Faculty of Human and Health Sciences, University of Bremen, Bremen, Germany; 4 Praxis für Kinder- und Jugendmedizin Roland Tillmann, Ärztenetz Bielefeld, Bielefeld, Germany; 5 Medical Faculty Mannheim, Center for Preventive Medicine and Digital Health (CPD), Heidelberg University, Mannheim, Germany

**Keywords:** Antibiotic prescription, Outpatient antibiotic prescriptions, Germany, Theoretical domains framework, Regional differences

## Abstract

**Background:**

Previous studies have identified substantial regional variations in outpatient antibiotic prescribing in Germany, both in the paediatric and adult population. This indicates inappropriate antibiotic prescribing in some regions, which should be avoided to reduce antimicrobial resistance and potential side effects. The reasons for regional variations in outpatient antibiotic prescribing are not yet completely understood; socioeconomic and health care density differences between regions do not fully explain such differences. Here, we apply a behavioural perspective by adapting the Theoretical Domains Framework (TDF) to examine regional factors deemed relevant for outpatient antibiotic prescriptions by paediatricians and general practitioners.

**Methods:**

Qualitative study with guideline-based telephone interviews of 40 prescribers (paediatricians and general practitioners) in outpatient settings from regions with high and low rates of antibiotic prescriptions, stratified by urbanity. TDF domains formed the basis of an interview guide to assess region-level resources and barriers to rational antibiotic prescription behaviour. Interviews lasted 30–61 min (M = 45 min). Thematic analysis was used to identify thematic clusters, and relationships between themes were explored through proximity estimation.

**Results:**

Both paediatricians and general practitioners in low-prescribing regions reported supporting contextual factors (in particular good collegial networks, good collaboration with laboratories) and social factors (collegial support and low patient demand for antibiotics) as important resources. In high-prescribing regions, poor coordination between in-patient and ambulatory health services, lack of region-level information on antimicrobial resistance, few professional development opportunities, and regional variations in patient expectations were identified as barriers to rational prescribing behaviour.

**Conclusions:**

Interventions targeting professional development, better collaboration structures with laboratories and clearer and user-friendly guidelines could potentially support rational antibiotic prescribing behaviour. In addition, better networking and social support among physicians could support lower prescription rates.

**Supplementary Information:**

The online version contains supplementary material available at 10.1186/s12913-024-11059-z.

## Background

Antimicrobial resistance is a major threat to global health systems [1]. Despite improvements in international surveillance programs [2], for example in the WHO European region in 2019 alone, around 541,000 deaths were associated with and 133,000 deaths were directly attributable to antimicrobial resistance [3]. One of the key drivers of antimicrobial resistance in humans is previous exposure to antibiotics [4]. To reduce the development of antimicrobial resistance, improving rational antibiotic prescription practices (i.e. avoiding unnecessary prescriptions) is crucial [5, 6]. Most antibiotic prescriptions in outpatient settings in Europe are for respiratory and urinary tract infections [7, 8]. In Germany, the setting of this study, most outpatient prescriptions for antibiotics are issued by general practitioners and paediatricians [8, 9].

Germany consistently ranks among the European countries with the lowest community consumption of antibiotics, for example, in the 2021 surveillance report of the European Centre for Disease Prevention and Control [10], Germany has the 3rd lowest community consumption of antibiotics for systemic use. Still, there is considerable regional variation in outpatient prescription rates across regions in Germany [11]. International research suggests that such regional differences in outpatient prescriptions cannot be fully explained by regional differences in infectious disease prevalence [12]. Instead, socioeconomic, demographic and cultural differences have been highlighted as additional key determinants [13].

A recent small-area analysis based on health insurance claims data [11], breaking down differences between the 401 administrative districts in Germany, found up to 4-fold differences in outpatient prescription rates for children (between 188 and 710 age- and sex-standardized outpatient prescriptions per 1000 persons/year), and more than 2-fold differences in adults (between 300 and 693 prescriptions per 1000 persons/year). These substantial regional variations in prescription rates continue to raise concern about the appropriateness of antibiotic prescribing practices in Germany [8].

At the same time, reasons for the observed regional differences in outpatient antibiotic prescription rates are not fully understood. On the one hand, urban-rural differences in prescription patterns might be due to differences in health care access and socioeconomic differences in populations such as age or deprivation status [14]. Proximity to animal breeding or fattening farms has also been associated with variations in antibiotic prescriptions [15]. Further regional differences exist in the quality and accessibility of out-of-hours emergency primary care settings, which have both been associated with an increase in antibiotic prescriptions [16].

On the other hand, non-clinical factors such as demographic and socioeconomic differences [13], or differences in patient demand and prescription practices have been suggested to underlie regional variations [17], and the influence of patient demand on inappropriate antibiotic prescriptions is well documented [18, 19]. Supporting small-area differences, calls have been made to take into account small-area regional factors in devising targeted interventions to support rational prescription practices [20].

Together, this suggests that a better understanding of the reasons underlying regional variations in outpatient antibiotic prescriptions is vital, especially for the development and implementation of better interventions to avoid inappropriate antibiotic prescriptions. The present study is based on a mixed methods research project commissioned by the German Federal Ministry of Health (SARA; “Studie zur Analyse der Regionalen Unterschiede bei der Antibiotika-Verordnung” [Study to analyse regional variations in antibiotic prescriptions]). Previous publications from this research project include the abovementioned small-area analysis of health insurance claims data [11] and a conference presentation containing some of the present data [21]. The current study focuses on the qualitative part of the project and reports results from interviews with prescribers in outpatient settings.

To this end, it builds on the patterns of regional differences identified in the previous quantitative study [11] to better understand the drivers of these regional differences in prescription behaviour based on perceptions of prescribers (general practitioners and paediatricians) in districts differing by outpatient antibiotic prescription rates.

In order to do so, an established framework of determinants of health care professional behaviours, the Theoretical Domains Framework (TDF; [22, 23]) was used to guide qualitative interviews with prescribers.

The TDF is a psychological model developed for healthcare and behaviour change research and is based on comprehensive reviews of behavioural theories [22, 24]. It comprises 14 key individual, social and contextual domains influencing human behaviour: knowledge, skills, social/professional role/identity, beliefs about capabilities, optimism, beliefs about consequences, reinforcement, intentions, goals, memory/attention/decision processes, environmental context/resources, social influences, emotion, and behavioural regulation. Both main effects of and interactions between domains are possible.

The TDF has been instrumental in examining individual determinants of antibiotic prescribing behaviour [25–28], and most studies show the domain of environmental context and resources to be influential for antibiotic prescriptions. However, which contextual aspects are particularly relevant is poorly understood to date.

The degree to which contextual resources and barriers as well as their interactions are specific to small-area districts and regions is vital to understand the observed variations in prescription rates and improve future intervention efforts. This study will therefore apply the TDF to understand differences in contextual determinants of antibiotic prescriptions and map these onto established small-area differences in paediatricians and general practitioners in Germany.

## Methods

### Participants and procedure

To identify region-level determinants of differences in outpatient antibiotic prescribing, semi-structured interviews were conducted with general practitioners and paediatricians working in outpatient settings. The protocol for this study was approved by the University of Bremen Ethics committee (AZ 2021-03).

### Data collection materials

An interview guide (supplementary file 1) based on the Theoretical Domains Framework (TDF) [22, 23] and previous studies using the TDF in antibiotic prescription contexts [25, 28] was designed with input from a paediatrician (RT) and pharmacoepidemiologists (UH, OS) and was pilot-tested with GP representatives known to the researchers. The interview guide started with informing participants about the status of their district as high-or low-prescribing and subsequently asked an open question on prescribers’ ideas on reasons for this. Following this, we asked prescribers for their perceptions on regional levels of TDF domains relevant for prescribing antibiotics [25, 28]; (i) knowledge, (ii) social support, (iii) environmental context and resources (and perceived differences to other districts), (iv) social and professional role, (v) social influences (patients), (vi) goals, (vii) beliefs about capabilities (patient expectation management), (viii) beliefs about consequences, (ix) optimism, (x) intentions, (xi) memory and attention processes.

### Recruitment

We employed purposive sampling and stratified potential participants based on our previous quantitative analysis of regional differences in medical claims data of outpatient antimicrobial prescriptions in Germany [11]. Here, differences in prescriptions were compared between administrative districts (“Landkreise” or “kreisfreie Städte”; Nomenclature of Territorial Units for Statistics NUTS level-3 subdivision [29]).

In order to compare and contrast health care providers’ perspectives on regional differences, we selected, separately for paediatricians and GPs, 5 districts each that were within the 5% highest antibiotic prescription rates per 1,000 insured persons, and 5 districts that were within the 5% lowest antibiotic prescription rates. Within each district group, we further selected rural and urban districts (classification based on official regional statistics in Germany; [30]) to account for potential differences in settlement structure.

Contact information for paediatrician and GP practices in the respective districts were obtained through the regional representations of the respective medical councils, and were contacted through email and phone calls. Snowball recruitment was used during which participants recommended further colleagues within the respective districts, and a total of 1,444 contact attempts were made. Participants received €75 (approximately US$80) for their participation.

### Procedure

Prescribers who had expressed interest in the study were emailed a participant information sheet and were asked to suggest a date and time for a phone interview. Semi-structured telephone interviews were subsequently conducted by experienced female and male qualitative researchers (CJ, BS, PK), audio recorded and were transcribed verbatim. Interviews lasted a mean of 45 min (range 30–61 min) and started with an introduction, brief overview of the study goals, and verbal informed consent was obtained prior to interview commencement. The interviewed prescribers had no personal or professional connection to the researchers before the interviews.

### Data analysis

Starting with the TDF domains in the interview guide, data analysis utilized an deductive approach and was based on thematic analysis [31]. Two researchers (CJ, BS) independently coded the material using MaxQDA data management software. Initial codes were reviewed between the two researchers, and saturation was achieved with both the paediatrician and GP interviews. All codes were mapped onto at least one of the TDF domains. Relationships between codes were examined looking at code overlaps in coded segments and analysing the relative proximity of coded segments in the transcribed text. The more frequently two codes appear in the same segment or in relative proximity, the more substantial overlaps between the codes are assumed. The relative positions of codes in this two-dimensional space were operationalized using multidimensional scaling implemented in MaxQDA. Here, a solution is estimated which replicates the distance between elements in the two-dimensional space between codes as well as possible relationships. Assigning of a code to a cluster of codes is estimated using the Unweighted Average Linkage method [32]. Disagreements were resolved through discussion between the researchers.

## Results

Results of the thematic analyses are presented separately for GPs and paediatricians.

### Participants

A total of 40 interviews (17 paediatricians; 10 from high-prescription and 7 from low-prescription districts, 23 GPs; 10 from high-prescription and 13 from low-prescription districts) were conducted. Participants had between 1 and 35 years of experience in their current positions (mean 13.4 years, SD 9.9 years). Interviews lasted an average of 44.8 min (SD 7.1 min, range 30–61 min).

### Paediatricians

**Fig. 1:**
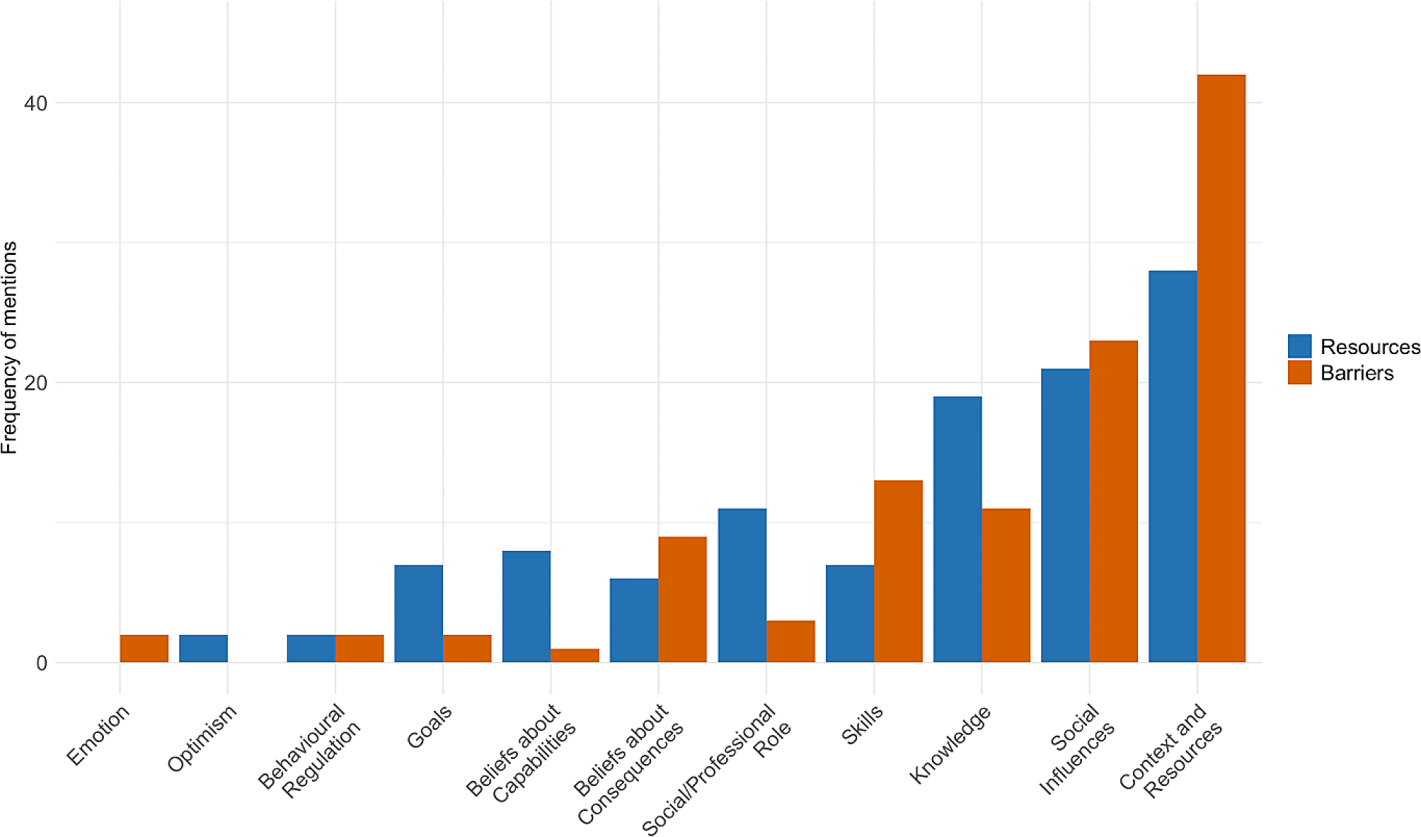
TDF domains mentioned as barriers (red) or resources (blue) by paediatricians

TDF domains on region levels mentioned as influencing paediatricians’ prescribing behaviour (Fig. 1) included context and resources (86 mentions), social influences (56 mentions), knowledge (36 mentions), skills (22 mentions), social/professional role (15 mentions), beliefs about consequences (15 mentions), beliefs about capabilities (9 mentions), goals (9 mentions), behavioural regulation (6 mentions), optimism (3 mentions) and emotions (2 mentions).

#### Context and resources

Regional context and resources can affect prescribing behaviour through multiple, direct and indirect pathways, according to the participating paediatricians. The distinction between contextual (i.e., factors specific to the region) and composition effects (i.e., factors resulting from the composition of the population within a region; [33]) is particularly relevant.

Paediatricians mainly mentioned contextual factors, e.g., air pollution as a risk factor:

*This area here is a former working-class area, air quality is poor, and this means we have more respiratory illnesses which are the most frequent reasons for antimicrobial prescriptions.*

(A, paediatrician, urban area, high prescription rate)

Similar direct contextual effects are evident in the density of paediatricians:

*…This means service provision for children in an emergency is limited, and they are rather seen by GPs. And the GPs are fantastic, […], but they don’t have our special training and might be a bit more anxious if they see a child with a high fever….*

(B, paediatrician, rural area, high prescription rate)

This low density then results in overload of the paediatricians, which in turn can increase antimicrobial prescriptions:

*I mean on a Monday in February I have seen about 200 children, or thereabouts. And then I can’t start discussing for ages, this just doesn’t work.*

(C, paediatrician, rural area, high prescription rates)

Suboptimal transition from in-patient to out-patient care were also seen to increase antimicrobial prescriptions in districts with higher prescription rates:

*…in the hospitals, they prescribe broad-spectrum antibiotics. And I have to say, after we have sat down together a year ago and have talked about outpatient antibiotic therapies, we had agreed on not prescribing some particular antibiotics. And now I see that these exact antibiotics are still being used in the hospital.*

(D, paediatrician, rural area, high prescription rates)

Contextual effects however also can constitute resources for lower prescription rates, for example in high-quality laboratories and quick turnaround times:

*This means we can get samples to them three or four times a day and are not dependent on pickups once a day like in the practices out there. This really is a resource I think*.

(E, paediatrician, rural area, low prescription rates)

#### Social influences

Social influences have been mentioned frequently, both as social influences through patients and through other health care providers. In particular where patient characteristics are being discussed, such influences could also be classified as compositional context resources (see above). However, as most of the quotes illustrate, these compositional factors also contain social influences.

Social influences as factors affecting high prescription rates are mainly located on patient level, illustrated in the following quote referring to patients with Middle-Eastern migration history:

*This is a totally different culture, also affecting ideas about illnesses. Their ideas are totally different, and antibiotics are seen as miracle drugs – they are over the moon if they can get an antibiotic.*

(F, paediatrician, urban area, high prescription rates)

However, the demand by patients is also being attributed to context effects such as dominating agricultural influences:

*I think that there are lots of expectations for antibiotics by patients. For example, I do have a mother who generally insists on getting an antibiotic for her child, and I wouldn’t prescribe it. And I tell you how she says it: ‘I also give this to my pigs, so it can’t be bad for my kids’. So I think that antibiotic practices in the farms around here, I think that this means they (antibiotics) are applied liberally and happily, and the parents have experience and want them for their kids as well.*

(C, paediatrician, rural area, high prescription rates)

At the same time, social influences are seen as malleable influences, in particular in combination with skills and knowledge which can then contribute to improvements in prescription practice:

*It has become much better, yes. They (patients) now understand it, they have gotten used to it. And now we have, when the doctor says, you don’t need an antimicrobial, then more than half of them don’t go and see another doctor immediately and say ‘I need an antibiotic’.*

(G, paediatrician, urban area, low prescription rates)

#### Knowledge

Knowledge included both information on current recommendations for antimicrobial prescribing, information on local resistance prevalence, information on local and personal prescription rates, and training content relevant to prescribing antimicrobials.

Participants from low-prescription districts mentioned knowledge on current recommendations as a resource and linked this knowledge to lower prescription rates within their districts:

*We feel quite well informed. And everyone builds on that through individual research, further training and talking to colleagues. And I think, else we wouldn’t see these numbers.*

(H, paediatrician, rural area, low prescription rates)

In contrast, paediatricians from high-prescription areas mentioned increased effort in obtaining relevant information:

*[…] There is no information in the district, you always have to look after this yourself.*

(I, paediatrician, rural area, high prescription rates).

In districts that had employed a paediatrician-initiated education programme (AnTiB; [34]), this programme was mentioned as an explicit resource:

*We used to have this little informal guideline here in (city), which is also lying around in out-of-hours paediatric services and which every paediatrician here is likely to have in their practice. It is very useful and if you are doing emergency shifts, you pull it out of the drawer, look at the dosage and then prescribe.*

(J, paediatrician, urban area, low prescription rate).

In contrast, the lack of specific knowledge in paediatric emergency services is seen as a barrier to effective prescribing:

*We live in one of the areas with the most children in Germany, and, you can’t make this stuff up, we don’t have a paediatric out-of-hours service. This means out-of-hours is staffed by colleagues, e.g., urologists who have no clue, who start googling first – and then quickly prescribe an antibiotic.*

(B, paediatrician, rural area, high prescription rate)

#### Skills

Skills as mentioned by the paediatricians include both discipline-specific and generic skills such as language skills or interpersonal skills.

Lack of specific treatment skills are mentioned as barriers to lower prescription rates by paediatricians in high-prescribing districts:

*Perhaps the experience that as a urologist, you might not have that much experience with these really high fever temperatures in toddlers under two years.*

(K, paediatrician, rural area, high prescription rate).

Similarly, a lack of language skills both on the side of the prescribers and patients is being seen as a barrier, both to non-prescribing and to instructing parents to monitor their children’s health:

*… there is such a large language barrier which prevents you from explaining what the parents have to look out for, what are the signs of deteriorations, when do they need to come back, well, that this is a problem overall.*

(L, paediatrician, urban area, high prescription rate)

#### Social and professional role

Social and professional role are mainly seen as a resource for low prescription rates. The main effects are seen to be indirect, via social norms and better professional networks. In some areas, this professional role is a relevant part of paediatricians’ identity which is used to be a role model to other paediatricians.

*I think there are these lighthouse or role model practices here, the bigger ones. And they do this on purpose, to set standards and blaze a trail, and the younger colleagues or others then orient themselves on them.*

(M, paediatrician, urban area, low prescription rates)

In addition, the social influence through networks is being seen as strengthened through social and professional roles and identity:

*So we do have quite a number of colleagues who are really well connected. They always participate in our quality groups, participate very reliably, and have good contact amongst themselves.*

(M, paediatrician, urban area, low prescription rates)

#### Beliefs about consequences

Beliefs about consequences tend to be related to contextual and environmental resources or barriers as well as regional outcomes. A particularly strong motive seems to be using antibiotics to prevent potential risks.

Paediatricians from districts with high prescription rates discuss avoiding consequences in particular with regards to patient overload:

*My personal record in winter was 209 children a day. […] I have briefly checked them and then prescribed an antibiotic, because even if most of it is viral, you have children with whooping cough and I tend to be generous, because the hospitals are full of pneumonia.*

(F, paediatrician, urban area, high prescription rates)

Paediatricians from districts with low prescription rates on the other hand discuss low beliefs about negative consequences such as patients changing doctors due to low competition pressure:

*So we don’t really have a competitive mindset here, because changes from one paediatrician to the other are really, really rare.*

(M, paediatrician, urban area, low prescription rates)

Interestingly, beliefs about consequences in terms of developing resistant microbes differ between paediatricians from low- and high-prescribing districts. Whereas those from high-prescribing districts argue that the responsibility for resistances is mainly located in the agricultural sector:

*I think that resistant microbes develop if the farms in the area use lots of antibiotics […] So the kids who have MRSA here, they are all from farms. So they didn’t get MRSA because we gave them antibiotics but because the farms at home use lots of antibiotics.*

(C, paediatrician, rural area, high prescription rates),

Those from low-prescription districts tend to attribute resistance development to health care professional behaviour:

*The less antibiotics one prescribes, and if this happens everywhere, then we can expect, that the development of resistances will be less bad than elsewhere.*

(H, paediatrician, rural area, low prescription rates)

#### Beliefs about competences

Beliefs about competences mainly revolved around perceptions of competence to influence local resistance developments and largely mirror those exemplified in the beliefs about consequences section.

#### Goals

Both paediatricians from low- and high-prescribing districts explicitly mentioned goals to prescribe less antimicrobials, and mention that these goals are also shared by colleagues in the respective districts. Differences exist in the context within goals are mentioned – paediatricians from low-prescription districts mention the goal of lower prescriptions as part of a combinations of goals (e.g., optimal therapy or limiting resistance development), paediatricians from high-prescription districts concentrate on potentially more relevant goals than lower prescription rates:

*…I think I can speak for most of my colleagues here, one tries to prescribe as little as possible. But if they really all read the reports, do they change their prescription behaviour, I doubt that. There are quite some other problems here that need solving as well.*

(F, paediatrician, urban area, high prescription rates)

#### Behavioural regulation

Behavioural regulation had only six mentions, but these were mainly together with contextual factors in districts with high prescription prevalence to highlight that contextual factors can pose barriers which also affect the low likelihood to change through impeding behavioural regulation:

*And I think that these are basically deeply rooted, historic, ritualized prescription patterns, which then manifest regionally such that it is really difficult to change this.*

(D, paediatrician, rural area, high prescription rates)

### General Practitioners (GPs)

**Fig. 2:**
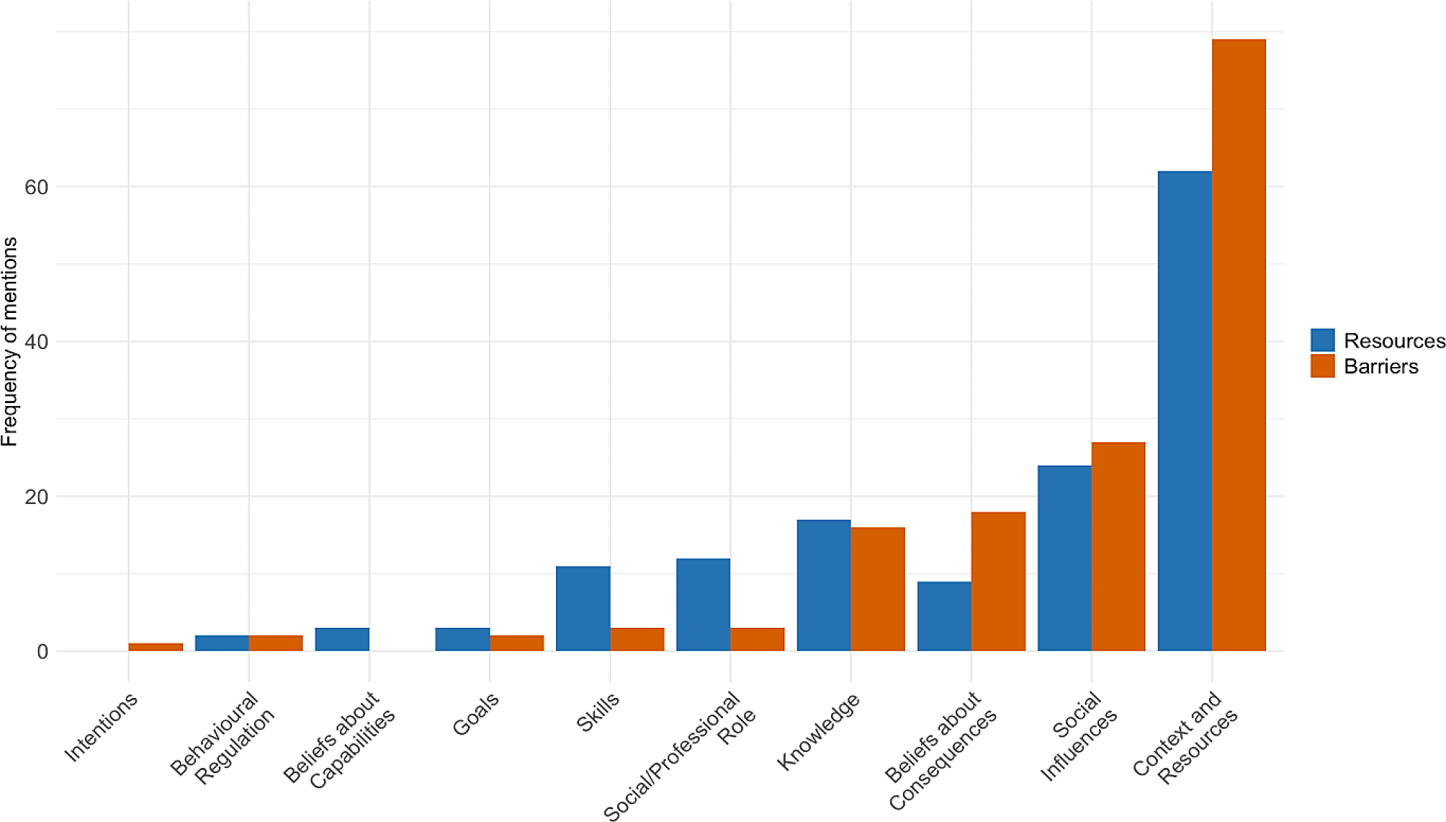
TDF domains mentioned as barriers (red) or resources (blue) by GPs

TDF domains on district level that affected GP prescribing behaviour (Fig. 2) included context and resources (159 mentions), social influence (60 mentions), knowledge (41 mentions), beliefs about consequences (29 mentions), social/professional role (16 mentions), skills (16 mentions), goals (6 mentions), and behavioural regulation (4 mentions).

#### Context and resources

Similar to the paediatric participants, GPs reported on a range of regional contextual factors that influenced prescribing behaviour. These can also be differentiated along contextual and compositional factors [33].

A combination of contextual (main industry in the region) and compositional (migrant workers in the main industry) is a good example for these influences:

*With the (migrant) workers in the meat industry, we do have a lot of people who might have potentially problems in dental hygiene, infections due to cuts for example. This happens a lot, and then increases the prescription of (antimicrobials).*

(N, GP, rural area, high prescription rates).

GPs also report on regional differences in the influence of pharmaceutical representatives in their practices. For example, a GP from a low-prescription rural district mentioned that their local quality circles “will not invite pharmaceutical representatives if possible”.

#### Social influence

Social influences differ between districts, according to GP participants, and similar to paediatricians, these influences come through colleagues and patients.

One example for a local social influence could be long established GPs who influence local quality circles:

*…as a young and newly arrived doctor, I quit going to the quality circles because the old guard was so present and influenced communication, work and thinking about practices. However, we do have now a new generation of GPs and things change.*

(O, GP, rural area, low prescription rates)

Patient-level influences are also perceived to differ between districts, with some of the differences in expectations to be prescribed antibiotics being attributed to cultural factors:

*There is a group of patients who are really eager to get antibiotics and who are incredibly demanding. Germans from the former Soviet Republics, and we do have many of them in this district. For them, it (not being prescribed antibiotics) is not a real therapy, even if it is viral….*

(P, GP, rural area, high prescription rates)

Similar to cultural factors, the age distribution in a district is perceived to affect prescription, with more older adults in a district being associated with higher antibiotic demand.

Similar to this influence on higher prescriptions, specific regional social influences are also perceived as being influential for low prescription rates:

*I mean, (city) is a very special city. It’s an administrative centre, a big university city, so I think there are a lot of people with a relatively high educational attainment, relatively little industry and I guess it’s also related to the fact that people have a bit of a different attitude.*.

(Q, GP, urban area, low prescription rates)

### Knowledge

Similar to the results in paediatricians, knowledge about current recommendations, information on local resistance, and training content relevant to prescribing antimicrobials were seen as relevant resources. One particular additional factor was that in one of the participating districts, the local university was seen as influential for particularly rational prescribing behaviour:

*I think that this is due to the fact that here in (city) there are many doctors who have studied in (city). And I remember from my studies that antibiotic prescriptions were an important topic, and that in microbiology et cetera we were always being reminded that one does not just prescribe antibiotics but needs to justify this really well.*

(R, GP, rural area, low prescription rates)

At the same time, similar to the paediatricians, a lack of knowledge in out-of-hours services is seen as a relevant factor for high prescription rates:

*But there are many colleagues working in the out-of-hours primary care and doing GP tasks who have for example an anesthesia background, or something else from the hospital, they don’t know it any better.*

(N, GP, rural area, high prescription rates)

These knowledge factors interact with resources and barriers on context level.

#### Beliefs about consequences

Similar to paediatricians, beliefs about consequences include beliefs about having to avoid liabilities, which are often mentioned in combination with structural and contextual factors:

*And if something does go wrong, and that’s always a problem in outpatient settings, you are the one who screwed it up. That’s what all the colleagues are afraid of. So the fear of making a mistake and not prescribing the antibiotic is always bigger than the fear of damaging something with the antibiotic.*

(S, GP, rural area, high prescription rates)

Losing patients to other practices in situations with strong competition was a strong belief about consequences in districts with high prescriptions:

*You can say, No I am not going to prescribe this, but then you lose the patient, they are just going somewhere else.*

(T, GP, urban area, high prescription rates).

At the same time, a lack of such perceived consequences has been perceived as a resource for lower prescriptions:

*…at least we don’t have to bow to patient demands too much. It is very different here compared to (city) where I was before, in the inner city, where there was a lot of competition due to too many GPs. You are much more likely to give in to irrational demands then.*

(U, GP, rural area, low prescription rates)

#### Social / professional role

Specific regional ideas on the professional roles are perceived to influence prescription behaviour, in particular in combination with specific aspects of rurality that could affect the composition of the local GP structure:

*I just see what kind of colleagues – to say it cautiously – are coming to this region, who take over old practices or establish new ones. They are not necessarily the most committed doctors.*

(V, GP, rural area, high prescription rates)

### Skills

In districts with low prescription rates, skills were mainly being mentioned with regards to interpersonal skills regarding expectation management with patients, which were perceived to be higher in the respective districts:

*…in fact, skills training in multiple areas. General communication skills, difficult patients, bad prognosis, diagnosis, or making the patients understand why a particular therapy is indicated – these are all key skills and have always been emphasized during our studies.*

(R, GP, rural area, low prescription rates)

#### Goals

Similar to paediatricians, GPs from both low- and high-prescription districts mention the goal of low prescription rates, and assume that their colleagues in the district have similar goals. GPs in low-prescription districts mention this goal as part of multiple goals (ideal therapy, avoid resistance) in low-prescription districts, GPs in high-prescription districts mention this goal as having lower priority compared to competing demands.

#### Behavioural regulation

Similar to the paediatricians, a lack of behavioural regulation in combination with contextual measures such as relatively old GPs in the district was seen as a risk factor for higher prescriptions:

*Prescription behaviour by older colleagues plays a role I think. You can see this when you look at the age structure of the GPs here. They tend to prescribe antibiotics quickly whenever there are respiratory infections.*

(W, GP, rural area, high prescription rates)

## Discussion

This study examined prescribers’ perceptions of region-specific drivers of outpatient antibiotic prescriptions. We conducted 40 interviews in districts stratified by antibiotic prescription rates, and mapped these perceptions on dimensions of the Theoretical Domains Framework [22, 23]. A total of 11 domains were identified, and these served as, partially interacting, barriers against and resources for low antibiotic prescription rates. Most barriers and facilitators were similar between paediatricians and GPs. However, while GPs mentioned the age and workforce structure in districts as additional barrier, paediatricians emphasized a lack of skills and knowledge of GP colleagues treating young children as a barrier in districts with only few paediatricians.

We could link differences in the perception of TDF domains and their interactions to differences in prescribing behaviour in the districts to identify overarching barriers and resources for appropriate prescription practices.

### Overarching barriers to low prescription rates

Both paediatric and GPs mentioned a lack of *knowledge* on district-level resistance developments as particular barrier to rational prescribing. This knowledge factor overlaps with a lack of contextual and environmental resources which could provide this information such as routine information flows between laboratories and health care providers.

Similarly, a lack of collaboration and coordination of knowledge in out-of-hours services was perceived to be associated with higher prescription rates – partly also due to a perception to avoid liabilities if prescribing antibiotics.

Lower health care provider density as *contextual* factor has been associated with higher prescription rates in previous international studies [35, 36]. In the present study, lower prescriber density has only indirectly been associated with higher prescription rates – in the cases where lower density correlates with suboptimal emergency services prescription guidelines [16].

Participants also associated specific regional industries in rural districts (pig farming and meat factories) with higher patient demands due to either antibiotic practices in farming [15] or an increased demand for antibiotics by migrant workers and due to cuts in meat factories.

*Social influences* included culture-specific expectations about the effectiveness of antibiotics leading to higher patient demand for antibiotics, which together with time pressure from high patient load increased pressure on prescribers during consultations. This finding replicates findings from other European studies on antibiotic prescribing behaviour [18, 19].

### Overarching resources for low prescription rates

Overarching resources for low prescription rates that were mentioned by both paediatricians and GPs included *environmental context and resources*. Here in particular existing local networks supporting quality control were perceived as supportive of appropriate prescribing, both through the provision of information, best-practice examples and social norms. This replicates an earlier study suggesting that well-functioning local or regional primary care networks in Germany are associated with more appropriate antibiotic prescribing [37]. In addition, laboratories routinely providing information on local resistance data were perceived as resources for rational prescribing, which is in line with previous studies in Germany outlining the lack of local resistance information as a barrier to appropriate prescribing [38] and, similarly, showing that practitioners perceive information on local resistance as beneficial [39]. Low local population demand for antibiotics was also perceived as resource, as participants reported this to positively impact their prescription practice.

### Implications

Most barriers and resources to rational outpatient prescribing in this study were contextual factors. However, contextual factors such as the local population, the main local branches of industry or (at least in Germany), or the free choice of practitioners to open practices anywhere within a district are not directly modifiable. This means that interventions should in particular target local collaboration structures and the availability of locally adapted guidelines.

If collaborations between local medical councils and laboratories can be improved to routinely provide local antimicrobial resistance data to prescribers, this information can readily be included into the prescription decision-making process [39]. In particular since both German and international studies [40] show that there is substantial variation in the degree to which individual practices take local resistance data into consideration, routine approaches are warranted. Germany has implemented a standardized surveillance program for multiresistant microbes such as MSRA [2], but the degree to which these surveillance findings are broken down locally and are available to practices varies considerably between districts, suggesting policies to standardise practice. If these findings are then included into local prescription guidelines such as the AnTiB guidelines [34], local prescription practices can be improved.

Routine antibiotic stewardship programmes that support paediatric and general practices could also help facilitating such closer collaborations and in turn build on some of the networking aspects mentioned as resources in the interviews. At the moment, antibiotic stewardship programs for outpatient settings in Germany are supported through national professional and scientific associations and are eligible for training credits, but implementation depends on local initiatives [5]. National policies to mandate such programmes would help to reduce the current regional disparities in antibiotic prescription practices, and the current antibiotic strategy of the German government DART 2030 [41] plans to explore compulsory training.

In terms of knowledge resources, participants mentioned that easy-to-use recommendations for emergency practice services are an important resource in particular if there is no paediatric emergency service, and children are seen by non-paediatrician practitioners. In Germany, initiatives such as Antibiotic Therapy in Bielefeld (AnTiB; [34]) provide such guidelines, but a systems-wide implementation of easy-to-follow guidelines such as e.g., NICE guidelines for upper respiratory tract infections [42] is currently lacking and would likely improve prescription practices in Germany.

Patient information such as leaflets might lead to increased patient knowledge about the role of antibiotics in managing infections and lower patient demand [43] without increased reconsultations [44]. At the same time, the role of involving audiences in the design of such leaflets and ensuring their understandability is crucial [45].

### Strengths and limitations

A particular strength of the study lies in using the TDF to examine district-level differences in prescription behaviour, which allowed us to identify and interpret the impact of the factors mentioned by GPs and paediatricians. This deductive approach allowed mapping key themes on an established framework, which can in turn be used to determine and develop potential intervention applications. Our study complements previous work applying the TDF to understand antibiotic prescribing behaviour [25] by extending the perspective of the TDF on individual determinants onto characteristics of the district.

At the same time, the perceptions of participants regarding district-level TDF-based characteristics are subjective perceptions and do not necessarily correspond to the actual level of resources and barriers in the districts. Compared to face-to-face interviews, telephone interviews miss out on nonverbal information, but have allowed us to accommodate prescribers’ schedules. Due to the self-report nature of interviews, demand characteristics might affect responses such that participants exaggerate or downplay relevant factors.

Saturation in that no new codes emerged was achieved in all study cells (defined by practitioner group, urban/rural practice site and prescription rates) apart from GPs from high-prescribing urban areas, where only one interview could be realised. It is thus possible that additional interviews could have provided additional barriers and resources.

## Conclusion

Substantial district-level differences in outpatient antibiotic prescriptions in paediatric and general practices can be mapped on differences in prescriber perceptions of district-level barriers and resources to rational prescribing. Given the regional variation in underlying reasons for inappropriate prescribing of antibiotics, similar qualitative studies in all districts in Germany with high prescription rates could be a promising approach to design targeted interventions. According to the results of interviews conducted in this study, routine provision of local antibiotic resistance data, better and clearer guidelines for paediatric patients in ambulatory emergency services, patient information and a wider implementation of standardised antibiotic stewardship programs could be promising targets for interventions.

### Electronic supplementary material

Below is the link to the electronic supplementary material.

Supplementary Material 1

Supplementary Material 2

## Data Availability

The qualitative data collected for this study was de-identified before analysis. Consent was not obtained to use or publish individual-level data from the participants and therefore may not be shared publicly. The de-identified (German) data can be obtained from the corresponding author upon reasonable request.

## References

[1] World Health Organisation. Antimicrobial resistance. https://www.who.int/health-topics/antimicrobial-resistance. Accessed 6 Oct 2023.

[2] Baede VO, David MZ, Andrasevic AT, Blanc DS, Borg M, Brennan G (2022). MRSA surveillance programmes worldwide: moving towards a harmonised international approach. Int J Antimicrob Agents.

[3] European Antimicrobial Resistance Collaborators (2022). The burden of bacterial antimicrobial resistance in the WHO European region in 2019: a cross-country systematic analysis. Lancet Public Health.

[4] Chatterjee A, Modarai M, Naylor NR, Boyd SE, Atun R, Barlow J (2018). Quantifying drivers of antibiotic resistance in humans: a systematic review. Lancet Infect Dis.

[5] Kern WV (2018). Rationale Antibiotikaverordnung in Der Humanmedizin. Bundesgesundheitsbl.

[6] O’Neill J. Tackling drug-resistant infections globally: final report and recommendations. 2016. https://amr-review.org/sites/default/files/160525_Final%20paper_with%20cover.pdf. Accessed 6 Oct 2023.

[7] Goossens H, Ferech M, Stichele RV, Elseviers M (2005). Outpatient antibiotic use in Europe and association with resistance: a cross-national database study. Lancet.

[8] Zweigner J, Meyer E, Gastmeier P, Schwab F (2018). Rate of antibiotic prescriptions in German outpatient care - are the guidelines followed or are they still exceeded?. GMS Hyg Infect Control.

[9] Poss-Doering R, Kronsteiner D, Kamradt M, Andres E, Kaufmann-Kolle P, Wensing M (2021). Antibiotic prescribing for acute, non-complicated infections in primary care in Germany: baseline assessment in the cluster randomized trial ARena. BMC Infect Dis.

[10] European Centre for Disease Prevention and Control (2022). Antimicrobial consumption in the EU/EEA (ESAC-Net) - Annual Epidemiological Report for 2021.

[11] Scholle O, Asendorf M, Buck C, Grill S, Jones C, Kollhorst B et al. Regional variations in Outpatient Antibiotic Prescribing in Germany: a small area analysis based on Claims Data. Antibiotics. 2022;11. 10.3390/antibiotics11070836 PMC9312140 35884090

[12] Zhang Y, Steinman MA, Kaplan CM (2012). Geographic Variation in Outpatient Antibiotic Prescribing among older adults. Arch Intern Med.

[13] Filippini M, Masiero G, Moschetti K (2006). Socioeconomic determinants of regional differences in outpatient antibiotic consumption: evidence from Switzerland. Health Policy.

[14] Devine P, O’Kane M, Bucholc M (2021). Trends, Variation, and factors influencing antibiotic prescribing: a longitudinal study in primary care using a Multilevel Modelling Approach. Antibiot (Basel).

[15] van der Roof I, Oude Boerrigter L, Wielders CCH, Smit LAM (2021). Use of antibiotics among residents living close to Poultry or Goat farms: a nationwide analysis in the Netherlands. Antibiot (Basel).

[16] Huibers L, Vestergaard CH, Keizer E, Bech BH, Bro F, Christensen MB (2022). Variation of GP antibiotic prescribing tendency for contacts with out-of-hours primary care in Denmark - a cross-sectional register-based study. Scand J Prim Health Care.

[17] Bizune D, Tsay S, Palms D, King L, Bartoces M, Link-Gelles R (2023). Regional Variation in Outpatient Antibiotic Prescribing for Acute Respiratory Tract infections in a commercially insured Population, United States, 2017. Open Forum Infect Dis.

[18] Sijbom M, Büchner FL, Saadah NH, Numans ME, de Boer MGJ (2023). Determinants of inappropriate antibiotic prescription in primary care in developed countries with general practitioners as gatekeepers: a systematic review and construction of a framework. BMJ Open.

[19] Lescure DLA, van Oorschot W, van der Brouwer R, Tjon-A-Tsien AML, Bonnema IV (2022). Providing antibiotics to immigrants: a qualitative study of general practitioners’ and pharmacists’ experiences. BMC Prim Care.

[20] Szymczak JE, Linder JA (2023). Cultural Variation in Antibiotic Prescribing: have Regional differences had their day?. Open Forum Infect Dis.

[21] Schüz B, Jones C, Scholle O, Haug U (2023). Regional variations in antibiotic prescribing in Germany: understanding differences through an adapted theoretical domains Framework. Health psychology for all: Equity, Inclusiveness and Transformation.

[22] Cane J, O’Connor D, Michie S (2012). Validation of the theoretical domains framework for use in behaviour change and implementation research. Implement Sci.

[23] Atkins L, Francis J, Islam R, O’Connor D, Patey A, Ivers N (2017). A guide to using the theoretical domains Framework of behaviour change to investigate implementation problems. Implement Sci.

[24] Michie S, Johnston M, Abraham C, Lawton R, Parker D, Walker A (2005). Making psychological theory useful for implementing evidence based practice: a consensus approach. Qual Saf Health Care.

[25] Courtenay M, Rowbotham S, Lim R, Peters S, Yates K, Chater A (2019). Examining influences on antibiotic prescribing by nurse and pharmacist prescribers: a qualitative study using the theoretical domains Framework and COM-B. BMJ Open.

[26] Bursey K, Hall A, Pike A, Etchegary H, Aubrey-Bassler K, Patey AM (2022). Physician-reported barriers to using evidence-based antibiotic prescription guidelines in primary care: protocol for a systematic review and synthesis of qualitative studies using the theoretical domains Framework. BMJ Open.

[27] Talkhan H, Stewart D, McIntosh T, Ziglam H, Abdulrouf PV, Al-Hail M (2022). Investigating clinicians’ determinants of antimicrobial prescribing behaviour using the theoretical domains Framework. J Hosp Infect.

[28] Sargent L, McCullough A, Del Mar C, Lowe J (2017). Using theory to explore facilitators and barriers to delayed prescribing in Australia: a qualitative study using the theoretical domains Framework and the Behaviour Change Wheel. BMC Fam Pract.

[29] Commission Regulation (EU). 2016/2066 of 21 November 2016 amending the annexes to Regulation (EC) No 1059/2003 of the European Parliament and of the Council on the establishment of a common classification of territorial units for statistics (NUTS). 2016.

[30] Laufende Raumbeobachtung - Raumabgrenzungen. BBSR. https://www.bbsr.bund.de/BBSR/DE/forschung/raumbeobachtung/Raumabgrenzungen/deutschland/kreise/siedlungsstrukturelle-kreistypen/kreistypen.html. Accessed 2 Oct 2023.

[31] Braun V, Clarke V (2006). Using thematic analysis in psychology. Qualitative Res Psychol.

[32] Kuckartz U, Rädiker S (2019). Analyzing qualitative data with MAXQDA: text, Audio, and video.

[33] Leyland AH, Groenewegen PP, Context, Leyland AH, Groenewegen PP (2020). Composition and how their influences vary. Multilevel Modelling for Public Health and Health Services Research: Health in Context.

[34] Bornemann R, Tillmann R (2019). [Antibiotic therapy in Bielefeld (AnTiB)-a local project for the promotion of rational antibiotic prescribing in the outpatient paediatric sector]. Bundesgesundheitsblatt Gesundheitsforschung Gesundheitsschutz.

[35] Stedman M, Lunt M, Davies M, Fulton-McAlister E, Hussain A, van Staa T (2020). Controlling antibiotic usage—A national analysis of General Practitioner/Family Doctor practices links overall antibiotic levels to demography, geography, comorbidity factors with local discretionary prescribing choices. Int J Clin Pract.

[36] Simon M, Thilly N, Pereira O, Pulcini C. Factors associated with the appropriateness of antibiotics prescribed in French general practice: a cross-sectional study using reimbursement databases. Clinical Microbiology and Infection. 2022;28:609.e1-609.e6. 10.1016/j.cmi.2021.08.026 34500079

[37] Poss-Doering R, Kamradt M, Glassen K, Andres E, Kaufmann-Kolle P, Wensing M (2020). Promoting rational antibiotic prescribing for non-complicated infections: understanding social influence in primary care networks in Germany. BMC Fam Pract.

[38] Neugebauer M, Ebert M, Vogelmann R (2019). [Lack of information and provision of information at the workplace as potential reasons for inappropriate antibiotic therapy in Germany]. Z Evid Fortbild Qual Gesundhwes.

[39] Petruschke I, Stichling K, Greser A, Gagyor I, Bleidorn J (2022). [The general practitioner perspective of a multimodal intervention for the adequate use of antibiotics in urinary tract infection - a qualitative interview study]. Z Evid Fortbild Qual Gesundhwes.

[40] McKay R, Law M, McGrail K, Balshaw R, Reyes R, Patrick DM. What can we learn by examining variations in the use of urine culture in the management of acute cystitis? A retrospective cohort study with linked administrative data in British Columbia, Canada, 2005–2011. PLoS One. 2019;14:e0213534. 10.1371/journal.pone.0213534 PMC6407775 30849104

[41] Bundesministerium für Gesundheit. DART 2030 - Deutsche Antibiotika-Resistenzstrategie. https://www.bundesgesundheitsministerium.de/themen/praevention/antibiotika-resistenzen/dart-2030. Accessed 1 Mar 2024.

[42] Kim NN, Marikar D (2020). Antibiotic prescribing for upper respiratory tract infections: NICE guidelines. Archives Disease Child - Educ Pract.

[43] de Bont EGPM, Alink M, Falkenberg FCJ, Dinant G-J, Cals JWL (2015). Patient information leaflets to reduce antibiotic use and reconsultation rates in general practice: a systematic review. BMJ Open.

[44] O’Sullivan JW, Harvey RT, Glasziou PP, McCullough A (2016). Written information for patients (or parents of child patients) to reduce the use of antibiotics for acute upper respiratory tract infections in primary care. Cochrane Database Syst Rev.

[45] Biezen R, Ciavarella S, Manski-Nankervis J-A, Monaghan T, Buising K (2023). Addressing Antimicrobial stewardship in primary care-developing patient information sheets using Co-design Methodology. Antibiot (Basel).

