# Supplementary material for: Drivers of district-level differences in outpatient antibiotic prescribing in Germany: a qualitative study with prescribers
Source: BMC Health Serv Res. 2024 May 6;24:589. doi: 10.1186/s12913-024-11059-z (PMC11075293; doi:10.1186/s12913-024-11059-z)
Supplement: Supplementary file 1 — Supplementary Material 1 [file 12913_2024_11059_MOESM1_ESM.docx]

**Interview Guide**

*Note: This guide has been translated from the original German version and may not capture all nuances in the original version.*

**Introductory question:**

What could be the reasons why *doctors* in XY prescribe antibiotics comparatively rarely / comparatively frequently? (independent of your personal prescription practice)

*TDF-based questions*

1. How well informed are *doctors in XY about important aspects of prescribing antibiotics? (e.g. about current guidelines or recommendations, alternative prescribing approaches, the local resistance situation) Follow-up Question: What is the reason for this?
2. Are there or have there been further training courses, quality circles or collegial exchanges in XY that have an impact on the prescribing practice of antibiotics in XY?
3. Which regional conditions - e.g. of a structural nature (e.g. contact to / accessibility of laboratories; transitions from inpatient to outpatient treatment) - could influence the prescription of antibiotics for *physicians in XY in individual cases?
4. How would you describe the attitude of *doctors* towards prescribing antibiotics in XY?
5. Do you have the impression that patients in your districts ask for more or less antibiotic prescriptions than elsewhere?
6. Do you think doctors in XY are more or less willing to issue prescriptions-in-case (“Bedarfsrezepte”) than elsewhere?
7. Do you need specific skills for patient communication in XY to change antibiotic prescription practices?
8. Do you think that doctors in XY have these skills?
9. How important is it to doctors in XY to delay the development of local antibiotic resistance?
10. Do you think the prescription behaviour of doctors in XY has an influence on the local antibiotic resistance development?
11. How optimistic are you that doctors in XY will only prescribe as many antibiotics as necessary in the future?

Final questions

1. Which factors influence your personal antibiotic prescription behaviour?
2. How could the situation in XY be improved?
