# Supplementary material for: Drivers of district-level differences in outpatient antibiotic prescribing in Germany: a qualitative study with prescribers
Source: BMC Health Serv Res. 2024 May 6;24:589. doi: 10.1186/s12913-024-11059-z (PMC11075293; doi:10.1186/s12913-024-11059-z)
Supplement: Supplementary file 2 — Supplementary Material 2 [file 12913_2024_11059_MOESM2_ESM.docx]

**Supplemental Table 1**

Consolidated criteria for reporting qualitative studies (COREQ): 32-item checklist

| **No** | **Item** | **Guide questions/description** | **Manuscript page/line** |
| --- | --- | --- | --- |
| **Domain 1: Research team and reflexivity** | | | |
| Personal Characteristics | | | |
| 1. | Interviewer/facilitator | Which author/s conducted the interview or focus group?  CJ and BS conducted interviews | 164-166 |
| 2. | Credentials | What were the researcher's credentials?  Authors have Master’s or PhD/MD degrees | 4-5 |
| 3. | Occupation | What was their occupation at the time of the study? | 164-166 |
| 4. | Gender | Was the researcher male or female?  Researchers were female (UH) and male (BS, OS, RT, CJ). Interviewers were female (PK) and male (BS, CJ) | 164-166 |
| 5. | Experience and training | What experience or training did the researcher have?  Credentials indicate successful completion of academic education, during which researchers have received training in qualitative methods | 4-5 |
| Relationship with participants | | | |
| 6. | Relationship established | Was a relationship established prior to study commencement?  No relationship was established prior to study commencement, participants were recruited via regional medical councils | 266 |
| 7. | Participant knowledge of the interviewer | What did the participants know about the researcher?  Researchers identified themselves, indicated their credentials and disclosed the study goals at the beginning of the telephone interviews | 167-170 |
| 8. | Interviewer characteristics | What characteristics were reported about the interviewer/facilitator?  Researchers identified themselves, indicated their credentials and disclosed the study goals at the beginning of the telephone interviews | 167-170 |
| **Domain 2: study design** | | | |
| Theoretical framework | | | |
| 9. | Methodological orientation and Theory | What methodological orientation was stated to underpin the study?  The interview guide for the study was based on the Theoretical Domains Framework | 132-135 |
| Participant selection | | | |
| 10. | Sampling | How were participants selected?  Participants were selected based on purposive sampling | 145 |
| 11. | Method of approach | How were participants approached?  Participants were approached by email and phone | 156-160 |
| 12. | Sample size | How many participants were in the study?  40 (17 paediatricians; 10 from high-prescription and 7 from low-prescription districts, 23 GPs; 10 from high-prescription and 13 from low-prescription districts) | 190-194 |
| 13. | Non-participation | How many people refused to participate or dropped out? Reasons?  No participants dropped out. A total of 1,444 contact attempts were made, no reasons known for refusing to participate | 256-257 |
| Setting |  |  |  |
| 14. | Setting of data collection | Where was the data collected?  Data was collected through telephone interviews at a time and date selected by participants. No information on exact location is available | 159-160 |
| 15. | Presence of non-participants | Was anyone else present besides the participants and researchers?  No | n/a |
| 16. | Description of sample | What are the important characteristics of the sample?  A total of 40 interviews (17 paediatricians; 10 from high-prescription and 7 from low-prescription districts, 23 GPs; 10 from high-prescription and 13 from low-prescription districts) were conducted. Participants had between 1 and 35 years of experience in their current positions (mean 13.4 years, SD 9.9 years). | 190-194 |
| Data collection |  |  |  |
| 17. | Interview guide | Were questions, prompts, guides provided by the authors? Was it pilot tested?  Interview guide is provided as supplementary file. The guide was pilot-tested. | 132-135 |
| 18. | Repeat interviews | Were repeat interviews carried out? If yes, how many?  No repeat interviews were carried out | n/a |
| 19. | Audio/visual recording | Did the research use audio or visual recording to collect the data?  Data was audiorecorded and transcribed verbatim | 166 |
| 20. | Field notes | Were field notes made during and/or after the interview or focus group?  No field notes were made | n/a |
| 21. | Duration | What was the duration of the interviews or focus group?  Interviews lasted an average of 44,8 minutes (SD 7.1 minutes, range 30-61 minutes). | 193-194 |
| 22. | Data saturation | Was data saturation discussed?  Saturation was achieved with both pediatrician and GP interviews | 652-655 |
| 23. | Transcripts returned | Were transcripts returned to participants for comment and/or correction?  No | n/a |
| **Domain 3: analysis and findings** | | | |
| Data analysis |  |  |  |
| 24. | Number of data coders | How many data coders coded the data?  2 coders (BS/CJ) | 173-174 |
| 25. | Description of the coding tree | Did authors provide a description of the coding tree?  Codes were mapped on the TDF, the findings according to the TDF are presented in the manuscript | 196-201  418-421 |
| 26. | Derivation of themes | Were themes identified in advance or derived from the data?  Themes were identified a-priori through the TDF | 172-173 |
| 27. | Software | What software, if applicable, was used to manage the data?  MaxQDA | 174 |
| 28. | Participant checking | Did participants provide feedback on the findings?  No | n/a |
| Reporting |  |  |  |
| 29. | Quotations presented | Were participant quotations presented to illustrate the themes / findings? Was each quotation identified?  Yes, quotes are presented and identified with pseudonyms (letter) | Results section |
| 30. | Data and findings consistent | Was there consistency between the data presented and the findings?  Data presented are used to illustrate findings | Results section |
| 31. | Clarity of major themes | Were major themes clearly presented in the findings?  Major themes were presented according to TDF domains | Results section |
| 32. | Clarity of minor themes | Is there a description of diverse cases or discussion of minor themes?  No | n/a |
